# Dynamic Compressive Mechanical Behavior and Microstructure Evolution of Rolled Fe-28Mn-10Al-1.2C Low-Density Steel

**DOI:** 10.3390/ma15103550

**Published:** 2022-05-16

**Authors:** Hao Wu, Yan Tan, Abdul Malik, Yangwei Wang, Syed Zohaib Hassan Naqvi, Huanwu Cheng, Jiebin Tian, Xianming Meng

**Affiliations:** 1 China Automotive Technology & Research Center Co., Ltd., Tianjin 300162, China; wuhao@catarc.ac.cn (H.W.); tianjiebin@catarc.ac.cn (J.T.); mengxianming@catarc.ac.cn (X.M.); 2 School of Material Science and Engineering, Beijing Institute of Technology, Beijing 100081, China; 15200803879@163.com (Y.T.); chenghuanwu@bit.edu.cn (H.C.); 3 School of Mechanical Engineering, Dongguan University of Technology, Dongguan 523808, China; 4 Department of Electronics Engineering, University of Engineering and Technology, Taxila 47080, Pakistan; zohaib.naqvi@uettaxila.edu.pk

**Keywords:** low-density steel, dynamic compression, strain hardenability, planar glide, mechanical twinning, temperature rise

## Abstract

In this study, the quasi-static and dynamic compressive mechanical behavior of a rolled Fe-28Mn-10Al-1.2C steel (low-density) was investigated. X-ray diffraction, optical microscopy, electron backscattered diffraction and transmission electron microscopy were conducted to characterize the microstructure evolution. The results displayed that the steel has remarkable strain rate sensitivity and strong strain hardenability under high strain rate compression. Most specifically, the deformation behavior was changed with the increase in the strain rate. A feasible mathematical analysis for the calculation of stacking fault energies and the critical resolve shear stresses for twinning was employed and discussed the nucleation of the twinning. The microband-induced plasticity and twinning-induced plasticity controlled the deformation under high strain rate compression and provided a strong strain hardening effect. The higher mechanical response can increase the broad use of low-density steel in automobile applications.

## 1. Introduction

Low-density steel is a promising structural material and seeking the great attention of researchers due to its high strength and high elongation to failure (EF) [1,2,3]. Hitherto, the mechanical behavior and microstructure features of Fe-Mn-Al-C steels were extensively reported. For example, Kim et al. [4] reported that the deformation was mediated by the interaction of twinning and dislocation in Fe-30Mn-1Al-0.3C steel. Sato et al. [5] proposed that the addition of aluminum (Al) in Fe-20Mn steel suppressed the martensitic transformation and promoted the formation of mechanical twinning. Frommeyer et al. [6] investigated the mechanical behavior of different incorporated Al, manganese (Mn) and carbon (C) in iron (Fe) and reported an excellent combination of high strength (~700–1100 MPa) and elongation (60%). According to their analysis, the presence of κ-carbides was responsible for high strength and large ductility. Yoo et al. [7,8] recommended that microband-induced plasticity (MBIP) was responsible for high strength and large plasticity in Fe-28Mn-9Al-0.8C steel.

Notably, the stacking fault energy (SFE) is a significant parameter that determines the mode of deformation (martensitic transformation, twinning or dislocation gliding plastic deformation) during plastic deformation [9]. Transformation-induced plasticity (TRIP) associated with martensitic transformation can control the deformation when the SFE of the austenitic steel is less than 18 mJ/m^2^ [10]. In addition, twinning-induced plasticity (TWIP) is the dominant deformation mode when the SFE is between 18 mJ/m^2^ and 35 mJ/m^2^ [10]. Contrary, the deformation mechanism changes from TWIP to dislocation gliding for the SFE > 35 mJ/m^2^. Park et al. [7,8] revealed that the dominant deformation mechanism is planar gliding if the SFE > 70 mJ/m^2^. Byun [11] presented the theoretical values of critical resolved shear stresses (CRSS) for mechanical twinning, which increases with the increase in SFE. Park et al. [9] reported the experimental validation of the theoretical calculation of Byun in a high alloyed Mn steel (full austenitic). Therefore, different deformation mechanisms lead to different strengthening factors for high Mn steels [12,13,14,15]. The superior mechanical properties of TWIP steels with strong strain hardenability are mainly due to the fact that mechanical twins reduce the effective mean free path of dislocations [16]. In addition, the microstructure of deformed high Mn steel is mediated by planar gliding, which also causes high strain hardenability. The high-density dislocation wall structures formed by planar gliding are considered to be effective barriers to dislocation movement [15].

The deformation behavior of materials and mechanical response under high strain rate compression is different than quasi-static compressive mechanical behavior [17,18]. The components of the low-density steel can be subjected to high strain rate compression under stringent environments such as the collision of the vehicles (cars, busses, etc.) at high speed can produce a strain rate of 10^3^ to 10^5^ s^−1^. These components must endure the high strain rate compression, and their complex deformation behavior should be studied. However, best of our knowledge, the reports on the deformation behavior under dynamic compression of low-density steel are very few [19,20]. Moreover, the temperature rise can lead to different SFEs (either increase or decrease) during high strain rate compression [19,20,21], which can change the deformation behavior and strain hardening of the low-density steel. Therefore, it is needed to study the mechanical response and deformation behavior of the low-density steel under high strain rate compression.

In this work, the mechanical behavior of a rolled Fe-28Mn-10Al-1.2C steel (hereinafter referred to as low-density steel) was studied under quasi-static (0.001 s^−1^) and high strain rate compression (3030 s^−1^ to 5950 s^−1^). The quasi-static compression can provide the reference values, which can further assist in evaluating the difference between the stress–strain behavior at low (0.001 s^−1^) and high strain rate compression (3030 s^−1^ to 5950 s^−1^). The low-density steel provided high strain hardenability under both quasi-static compression and dynamic compression. The high strain hardenability and complex deformation behavior under high strain rate compression were further evaluated. The microstructure of the as-received specimen was studied through X-ray diffraction (XRD), optical microscopy (OM), electron backscattered diffraction (EBSD), transmission electron microscopy (TEM) and deformed specimens were studied by TEM.

## 2. Experimental

Firstly, the ingot of low-density steel was fabricated in a vacuum induction furnace. The composition is presented in Table 1. Then, it was homogenized in a furnace at a temperature of 1150 °C for 60 min. Further, it was subjected to a hot rolling process, and a plate of thickness ~12 mm was achieved. The area reduction ratio was ~80%. Finally, it was annealed at a temperature of 1100 °C for 60 min. The whole fabrication was carried out in Central Iron and Steel Research Institute, Beijing, China. Specimens of dimensions ϕ = 4 mm × 4 mm were machined along the normal direction (ND) of the sheet. The quasi-static compression tests were performed on an (Instron-5985, Norwood, MA, USA) at ambient temperature under a strain rate of 0.001 s^−1^. The flow stresses at true strains of 5%, 10%, 15%, 20% and 25% under a strain rate of 0.001 s^−1^ were considered as reference values. The dynamic compression tests in the strain rate range 3030 s^−1^ to 5950 s^−1^ were performed on a (split Hopkinson pressure bar (SHPB), Beijing, China). For accuracy, three tests were conducted for both quasi-static and dynamic compression.

For microstructure evolution, the rolled specimen was conducted on scanning electron microscopy (SEM, Fei Quanta 450F, Hillsboro, OR, USA) equipped with an EBSD system. The data acquisition was performed using (HKL, Channel-5 software, version 3.1). The step size and grid size for EBSD were set at 0.5 µm and 405 × 404, respectively. XRD, (Almelo, The Netherlands) analysis of as-received specimen was also conducted. For microstructure evolution of high strain rate compressed specimens, the TEM was conducted on Tecnai G^2^ F20 S-TWIN, Barcelona, Spain. For the preparation of specimens, initially, the samples with a diameter of 3 mm and a thickness of 1 mm were machined from the as-received and deformed specimens. Later, they were grounded by SiC abrasive papers and reduced the thickness to 30 µm. Further, the electro-polishing of deformed specimens was carried out by using a solution (perchloric acid and ethanol). Finally, the specimens were thinned by ion-milling (Leica RES 101, Barcelona, Spain) under the current 1 mA and time of 30 min.

## 3. Results and Discussions

**Figure 1:**
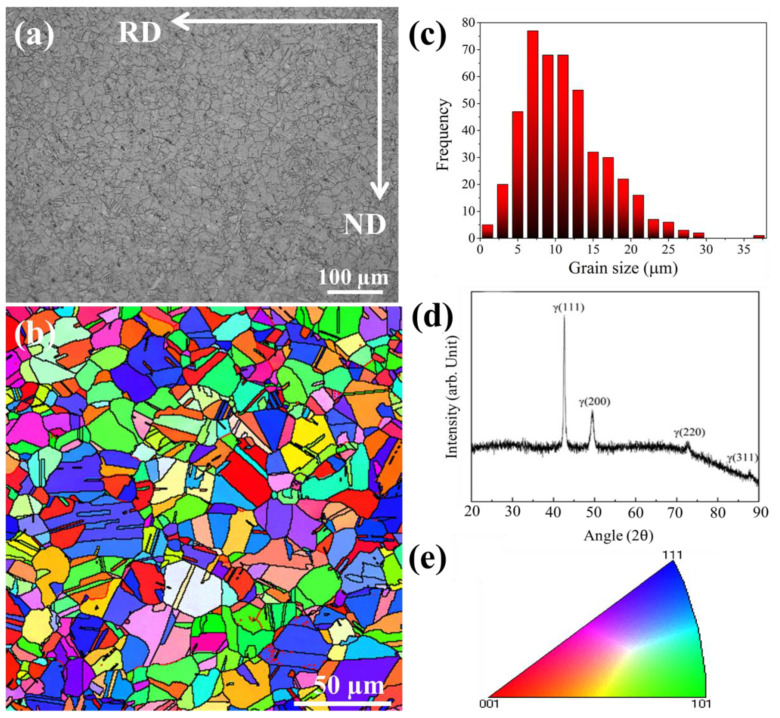
(**a**) OM of low-density steel along RD-ND plane; (**b**) IPF map of low-density steel along RD-ND plane; (**c**) grain size statistical analysis; (**d**) XRD of rolled steel; (**e**) legend map of corresponding the IPF map.

Figure 1 displayed the OM, IPF map and XRD of rolled low-density steel along RD-ND plane. It can be seen that the morphology of the grains is heterogeneous, i.e., comprised of coarse grains, equiaxed grains, some small grains and grains annealed twinning, as shown in Figure 1a,b. The average grain size is ~10 μm, as shown in Figure 1c.

Figure 1d represents the XRD of the as-received low-density steel. Notably, the peaks revealed that the microstructure is consisted of fully austenitic. The potential reason for fully austenitic formation is the addition of high content of Mn, which stabilized the austenite.

**Figure 2:**
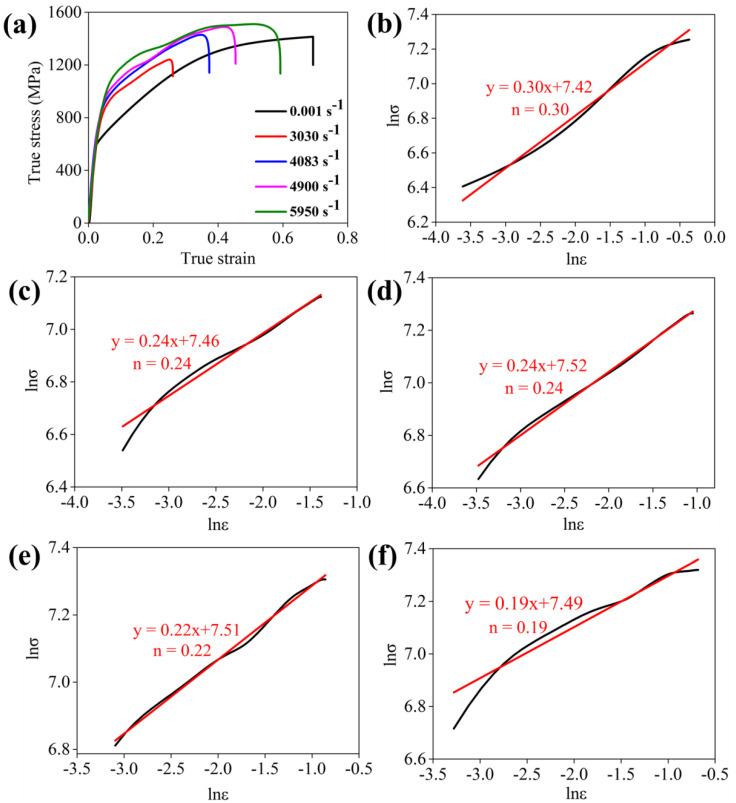
(**a**) Stress–strain curves under given conditions; (**b**–**f**) strain hardening exponent graphs at 0.001 s^−1^, 3030 s^−1^, 4083 s^−1^, 4900 s^−1^ and 5950 s^−1^, respectively.

The mechanical response under quasi-static and dynamic compression is provided in Figure 2a. It can be seen that low-density steel exhibited a superior combination of compressive yield strength (CYS) ~580 MPa, ultimate compressive strength (UCS) ~1400 MPa and EF 0.69. In contrast, the results of dynamic compression exhibited different mechanical behavior. The CYS, UCS and EF tend to increase with an increase in strain rate. Notably, the CYS of the high strain rate compressed specimens are higher than the quasi-static compressed specimen. The UCS reached 1450 MPa at a strain rate of 5950 s^−1^, which is slightly higher than the quasi-static UCS.

In order to evaluate the stress–strain behavior under both conditions, we have evaluated the strain hardening exponent (*n*-value), strain rate sensitivity (*m*-value) and adiabatic temperature rise. For evaluating the *n*-value, the following equation was employed.

(1) $$\sigma =K\varepsilon^{n}$$

Here, *σ* and *ε* represent true stress and true strain, respectively, *K* represents the strain hardening coefficient, and *n* is the strain hardening exponent. By taking the logarithm (ln) of Equation (1), we are arrived at;

(2) lnσ=nlnε+lnK

The value of *n* can be calculated by making a linear regression analysis of ln*σ* and ln*ε*. After thorough analysis, the *n*-values were evaluated and are shown in Figure 2b–f. The results provided that the *n*-value ~0.30 is highest in quasi-static compression compared to higher strain rates compression. This is well-matched with stress–strain curves. However, *n*-values are ≥0.19 and approach 0.24 under high strain rate compression, which also assists in understanding the high strain hardenability.

The strain rate sensitivity (*m*-value) [22] can be evaluated by using Equation (3) at different strains.

(3) $$m=(\frac{\partial \ln\bar{\sigma }}{\partial \ln\bar{\dot{\varepsilon }}})=(\frac{\partial \ln\frac{\sigma }{\sigma_{0}}}{\partial \ln\frac{\dot{\varepsilon }}{{\dot{\varepsilon }}_{0}}})≅\frac{\ln(\frac{\sigma }{\sigma_{0}})}{\ln(\frac{\dot{\varepsilon }}{{\dot{\varepsilon }}_{0}})}$$

**Figure 3:**
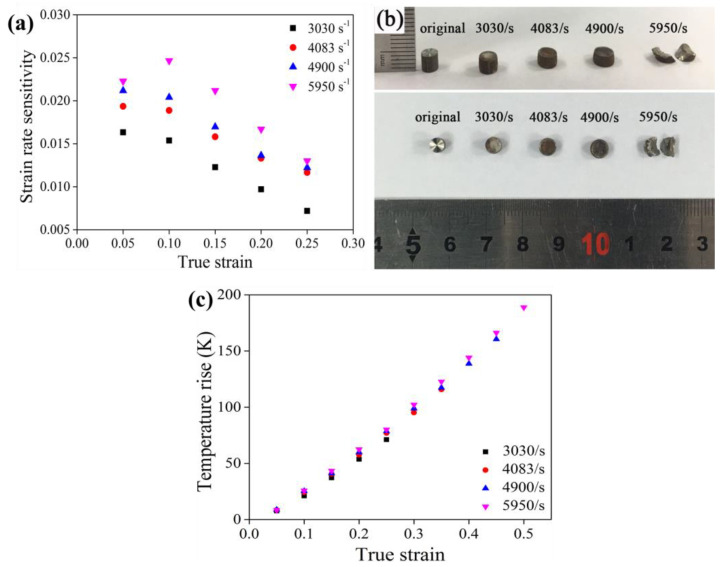
(**a**) Strain rate sensitivity at various strain rates as a function of true strain; (**b**) as-received and deformed specimens; (**c**) temperature rise under different strains.

Here $\bar{\sigma }$ and $\bar{\dot{\varepsilon }}$ are defined as the normalized stress and normalized strain rate, respectively; σ and $\dot{\varepsilon }$ represent flow stress and strain rate, respectively; $\sigma_{0}$ and ${\dot{\varepsilon }}_{0}$ represent reference stress and strain rate, respectively. The results of *m*-values are shown in Figure 3a. It can be inferred that the strain rate sensitivity declines slightly with the increase in the strain and displays a negligible difference at a strain of 0.25 under higher strain rates except for 3030 s^−1^. This behavior is common in metals and can be correlated with thermal softening due to adiabatic temperature rise in the specimen [23]. It is obvious that the temperature rise can dissipate quickly under quasi-static compression, whilst it cannot diffuse into the surrounding environment under high strain rate compression. Thereby, it has a significant impact on mechanical behavior. The temperature rise can be calculated through the following Equation (4).

(4) $$\Delta T=\frac{\eta }{\rho c}\int_{\varepsilon_{i}}^{\varepsilon_{i+1}}\sigma (\varepsilon_{i},{\dot{\varepsilon }}_{i},T_{i})d\varepsilon$$

where ρ represents the density (6.92g cm^−3^), and c represents the specific heat (0.5 Jg^−1^K^−1^) at room temperature, *η* is the fraction of the plastic work converted into heat and can be estimated to be 1.0 at high strain rates [24]. The estimated converted heat and temperature rise after dynamic compression are listed in Table 2.

In addition, the as-received and deformed specimens and temperature rise as a function of strains are shown in Figure 3b,c. The specimens were not broken until the strain rate was 4900 s^−1^. The heat and the temperature rise lead to the thermal softening and take part in overcoming the significant increase in UCS. This is the potential reason for decreasing the *n*-value under dynamic compression compared to quasi-static compression.

**Figure 4:**
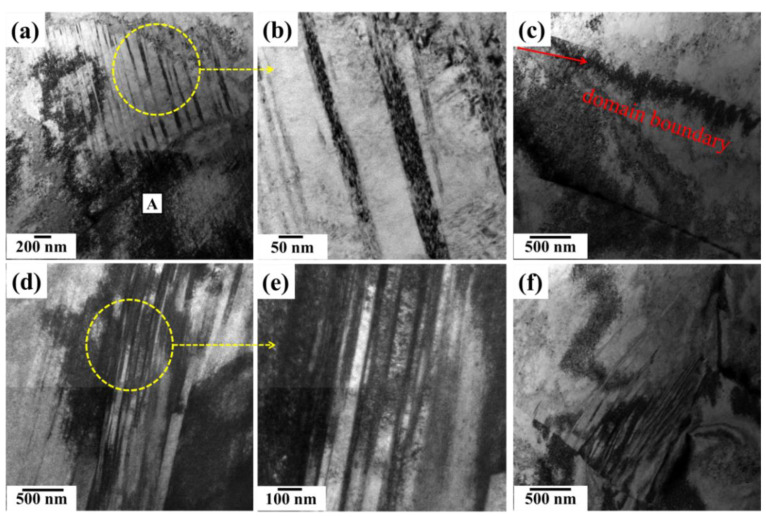
TEM representative micrographs of deformed specimens of low-density steel at various strain rates; (**a**–**c**) at 3030 s^−1^; (**d**–**f**) 4083 s^−1^.

For analyzing the microstructure and its correlation with the stress–strain response, TEM analysis was conducted, as shown in Figure 4. The long and straight dislocations aligned along two particular directions were observed at a strain rate of 3030 s^−1^ (Figure 4a), which is the characteristic of planar gliding. The spacing of dislocation structures was about 120 nm (Figure 4b). The low energy dislocation structure was also observed as marked ‘A’ in Figure 4a. Additionally, Figure 4c revealed single-walled TL domain boundaries (DBs). The formation of DBs is attributed to the misorientation increased by the rotation of TL domains [25].

With an increase in the strain rate up to 4083 s^−1^, the highly-dense dislocation walls (HDDWS) were formed by a large number of dislocations, as shown in Figure 4d. The spacing of dislocation was 30–80 nm, as shown in Figure 4e. In addition, we have also observed HDDWS terminated at the grain boundary (Figure 4f), which is the characteristic of planar slip [25,26,27]. Similar results were reported in the refs. [28,29,30].

**Figure 5:**
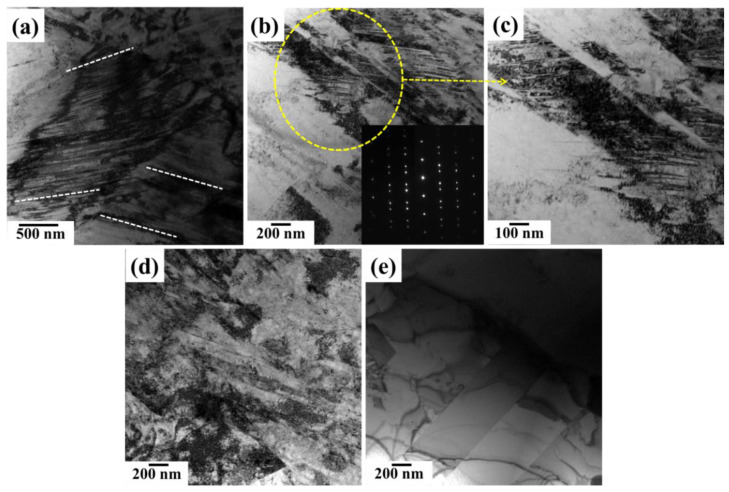
TEM representative micrographs of deformed specimens of low-density steel at various strain rates of (**a**–**c**) at 4900 s^−1^; (**d**) 5950 s^−1^; (**e**) as-received specimen.

Microbands (MBs) formed by a couple of dislocation walls were observed at the strain rate of 4900 s^−1^. These bands have subdivided the grains [28] and increased the strength of the steel, as shown in Figure 5a. In addition, the mechanical twins also appeared (Figure 5b). The magnified view of Figure 5b is shown in Figure 5c, which confirms that the mechanical twinning controlled the deformation with an increase in the strain rate. The SAED pattern (in Figure 5b) also confirmed the existence of mechanical twinning.

Multiple slips occurred under a strain rate of 5950 s^−1^, as shown in Figure 5d. The TL domains were further subdivided by increasing the number of either DB or MB intersections. For comparison, we conducted a TEM of the as-received specimen, which shows a low dislocation density (Figure 5e). The morphologies of both figures reveal that the dislocation was accumulated at grain boundaries or at coherent interfaces of twins under high strain rate compression.

In short, it can be concluded that the deformation mode was changed with the change in the strain rate. The main deformation mode is the combination of both planar gliding and mechanical twinning under high strain rate compression [7,8].

It is worth noting that the deformation mechanism of austenitic steels depends on the SFE, which is further related to composition and temperature [31]. A regular solution model [10,11,30,31,32] was employed, and the values of SFE and ΔGγ→ε of low-density steel at ambient temperature were calculated, which were found to be ~66 mJ/m^2^ and ~858 J/mol, respectively. Most specifically, the SFE can be varied for different strain rates due to temperature rise under dynamic compression; therefore, based on the regular solution model, we calculated the values of SFE (listed in Table 2). The value of SFE is much higher than 35 mJ/mol, which assists in understanding that the deformation mode is governed by the dislocation gliding [10] and is well consistent with this study. However, we also observed mechanical twinning. The CRSS for mechanical twinning is the stress required to separate the Shockley partials infinitely on the slip plane [11], which can be expressed as the following Equation (5).

(5) $$\sigma_{T}=2\frac{\Gamma }{b}$$

where Γ represents the SFE, *b* is the magnitude of the Burgers vector (0.147 nm) [11]. The SFE increased due to temperature rise during the dynamic loading. Therefore, based on Equation (5), it can be deduced that the CRSS for mechanical twinning would increase. The calculated value of SFE energy is 126 mJ/m^2^ at the strain rate of 4900 s^−1^, and the corresponding CRSS is ~1718 MPa. The flow stress at the strain rate of 4900 s^−1^ is lower than the corresponding CRSS, but the mechanical twins still appeared in the microstructure at the strain rate of 4900 s^−1^. The possible reasons for the nucleation of the mechanical twinning are the followings: (1) Equation (5) was derived without taking into account the strain rate effect. The CRSS for mechanical twinning decreases with the increase in the strain rate [30]. (2) Park [9] reported that the Fisher interaction (dislocation-short range order) decreases the CRSS for mechanical twinning in the FCC metals. Similarly, we observed the short-range order dislocation, which reduced the CRSS of mechanical twinning. Therefore, mechanical twinning is likely to occur when the stress is lower than the CRSS evaluated by Equation (5). The SFE increased due to temperature rise under dynamic compression, and the CRSS for mechanical twinning should further increase. However, dislocation gliding and an extremely short time of compression promoted the occurrence of mechanical twinning under high strain rates.

TL DBs, HDDWS and MBs are the characteristics of planar gliding and play an important role in the strain hardening of high Mn steels [7,8,29,30,31,32,33]. These fine dislocation substructures make the glide plane spacing very narrow. The dislocations, DBs and MBs, terminate at grain boundaries or twin boundaries, resulting in grain subdivision. The grain subdivision is another hardening factor that causes an excellent combination of strength and ductility of the present low-density steel [8]. In addition, the interaction of twinning and dislocation also increases the strength of the material [17,18,34,35,36]. Therefore, the synergistic effect of all these factors increased the UCS under high strain rate compression.

## 4. Conclusions

The microstructure evolution of high strain rate compressed low-density steel was studied, and it was further correlated with the quasi-static and dynamic mechanical behavior. The interesting outcomes are as follows:

1. The stress–strain curves revealed a positive strain rate sensitivity under all strain rates. The flow stresses were continuously increased with an increase in the strain rate at a fixed strain;
2. The low-density steel showed a strong strain hardening effect under quasi-static and dynamic compression. The strain hardening exponent decreases under dynamic compression due to the thermal softening effect caused by temperature rise;
3. The microstructure evolution of deformed specimens showed the highly-dense dislocation walls, domain boundaries and microbands under a high strain rate of 4083 s^−1^. With the increase in strain rate up to 4900 s^−1^, mechanical twinning was also nucleated. The dominant deformations at each strain rate are well consistent with the stacking fault energies calculations;
4. The strong strain hardening originated from plasticity induced by microbands and twins. The microband-induced plasticity and twinning-induced plasticity enhanced the mechanical properties of the steel under dynamic compression.

## Figures and Tables

**Table 1:** Chemical composition (wt.%) and density of Fe-28Mn-10Al-1.2C steel.

	Composition (wt.%)				Density (g cm^−3^)
	C	Mn	Al	Fe	
**Fe-28Mn-10Al-1.2C**	1.20	28.00	10.00	Bal.	6.92

**Table 2:** The estimated converted heat (ΔQ), the temperature rise (ΔT), and the stacking fault energy (SFE) increment for different strain rates after dynamic compression.

Strain Rate/s^−1^	ΔQ (J/cm^3^)	ΔT (K)	ΔSFE (mJ/m^2^)
3030	258	75	17
4083	432	125	47
4900	559	162	60
5950	780	226	81

## Data Availability

Not applicable.
